# From one to twelve: feasibility and clinical utility of deep learning-derived 12-lead ECGs for remote cardiac monitoring

**DOI:** 10.3389/fcvm.2026.1856637

**Published:** 2026-06-09

**Authors:** Haoyang Hu, Zekai Yu, Feiwei Qin, Fei Yang

**Affiliations:** 1 Department of Neurology, The First Medical Centre, Chinese PLA General Hospital, Beijing, China; 2 Medical School of Chinese PLA, Beijing, China; 3 School of Computer Science and Technology, Hangzhou Dianzi University, Hangzhou, Zhejiang, China

**Keywords:** deep learning, ECG reconstruction, multi-task learning, remote monitoring, transformer, wearable devices

## Abstract

**Background:**

Cardiovascular diseases (CVDs) remain the leading cause of global mortality. While the 12-lead electrocardiogram (ECG) is the clinical gold standard, wearable devices typically provide only single-lead monitoring (Lead I), which lacks the spatial resolution required to detect complex pathologies such as myocardial infarction or ST-segment changes.

**Objective:**

This study proposes a deep learning framework capable of reconstructing a full 12-lead ECG from a single-lead input while preserving diagnostic integrity.

**Methods:**

We developed TONet (Transformer-based Optimization Network), a multi-task framework evaluated on the PTB-XL dataset (*n* = 21,837). The architecture integrates a Convolutional Neural Network (CNN) encoder to extract local morphological features and a Transformer encoder to capture global temporal dependencies across the cardiac cycle. To ensure clinical validity, the model was trained using a dual-objective loss function, simultaneously optimizing for signal reconstruction (MSE) and multi-label diagnostic classification (BCE) across five diagnostic super-classes.

**Results:**

TONet achieved an overall Pearson Correlation Coefficient (PCC) reaching 0.673 on the independent test set. Clinical agreement assessment via Bland-Altman analysis revealed a negligible per-lead bias (|Bias| ≤ 0.0024 mV across all 11 reconstructed leads), indicating no systematic voltage drift. In terms of diagnostic utility, the reconstructed 12-lead signals achieved a macro-averaged AUROC of 0.821 (95% CI: 0.810–0.831), with per-class AUROCs of 0.874 (NORM), 0.770 (MI), 0.862 (STTC), 0.782 (CD), and 0.815 (HYP). To assess generalisability beyond a single cohort, we additionally performed an independent in-distribution validation on the Chapman-Shaoxing 12-lead ECG database (*n* = 43,559 quality-controlled recordings), where TONet, retrained from scratch using the identical protocol, achieved an overall PCC of 0.612 (95% CI: 0.552–0.666) and substantially outperformed the linear-regression baseline (PCC = 0.498), confirming that the reconstruction benefit transfers to a second, hardware- and population-distinct cohort.

**Conclusion:**

TONet demonstrates that a hybrid CNN–Transformer network with a multi-task reconstruction-plus-classification objective can recover morphologically faithful 12-lead surrogates from a single Lead I drawn from clinical PTB-XL recordings. We position this work as a regularized deep-learning framework for multi-lead reconstruction rather than a deployable clinical device: the reconstructed precordial leads are insufficient for fine-grained ST-elevation myocardial infarction (STEMI) screening, and external validation on true wearable recordings remains essential.

## 1 Introduction

Cardiovascular disease (CVD) remains the leading cause of death globally, claiming approximately 17.9 million lives annually and accounting for 32% of all global deaths (1). As a non-invasive modality for detecting cardiac electrical activity, the electrocardiogram (ECG) has long served as the gold standard tool for the screening, diagnosis, and monitoring of CVDs (2). By recording cardiac electrical activity from various spatial angles, the standard 12-lead ECG provides rich diagnostic information, including the localization of myocardial infarction (MI), the identification of ST-segment changes, and the detection of structural abnormalities such as ventricular hypertrophy (3).

In recent years, with the rapid advancement of wearable technology, smartwatches and portable ECG monitoring devices have become increasingly prevalent (4). These devices typically acquire only single-lead ECGs (predominantly Lead I). While demonstrating certain clinical value in arrhythmia screening and atrial fibrillation detection (5), their inherent limitations in spatial resolution preclude them from meeting more complex diagnostic requirements. Specifically, single-lead ECGs fail to capture electrical activity from different regions of the heart, consequently presenting significant deficiencies in critical clinical tasks such as identifying ST-Elevation Myocardial Infarction (STEMI), determining the site of infarction, and assessing voltage criteria (6). This technological gap fundamentally limits the potential application of wearable devices in the fields of telemedicine and home-based cardiac monitoring.

To bridge the gap between home monitoring and clinical diagnosis, researchers have begun to explore the feasibility of utilizing deep learning techniques to reconstruct full 12-lead ECGs from limited leads. Early studies primarily employed traditional Convolutional Neural Networks (CNNs) or Generative Adversarial Networks (GANs) for waveform reconstruction (7, 8). However, these methods suffer from inherent limitations in capturing the long-range temporal dependencies of ECG signals: CNNs are limited by the receptive field size of their convolutional kernels, making it difficult to effectively model global features spanning multiple cardiac cycles; conversely, while GANs excel at generating realistic waveforms, their training process is often unstable and lacks explicit constraints on clinically relevant diagnostic features (9).

Addressing these challenges, this study proposes a Transformer-based Optimization Network (TONet), designed to reconstruct a complete 12-lead ECG from a single Lead I input under the controlled setting of the PTB-XL clinical database. The main methodological elements of TONet are twofold; we note that neither the CNN–Transformer hybridization nor multi-task reconstruction-plus-classification is novel in isolation, and we frame our contribution as the systematic ablation, baseline comparison, and per-lead amplitude characterization of such a design under the single-Lead-I constraint:

First, a hybrid architecture design. TONet organically integrates a CNN with a Transformer encoder. The CNN module is responsible for extracting local morphological features (such as QRS complex morphology and T-wave characteristics), while the Transformer encoder captures the global temporal dependencies of ECG signals via a self-attention mechanism, including rhythm regularity across cardiac cycles and electrophysiological correlations between leads (10). This design enables the model to leverage both local and global information for waveform reconstruction.

Second, a multi-task learning strategy. Unlike traditional methods that pursue only pixel-level waveform reconstruction, TONet adopts a multi-task learning framework to simultaneously perform 12-lead waveform reconstruction and five-class diagnostic classification (including Normal [NORM], Myocardial Infarction [MI], ST/T Changes [STTC], Conduction Disturbance [CD], and Hypertrophy [HYP]). The core philosophy behind this design is that the diagnostic classification task serves as an auxiliary supervision signal, guiding the model to learn feature representations with clinical diagnostic significance rather than merely performing numerical fitting of the waveform (11). Consequently, the reconstructed waveforms are more likely to preserve pathological features critical for clinical decision-making.

In this study, we systematically trained and evaluated TONet on the PTB-XL dataset (*n* = 21,837) (12), and benchmarked it against (i) a population-mean floor, (ii) a Kors-style linear regression baseline, (iii) a single-lead-input classifier without the reconstruction branch, and (iv) CNN-only and Transformer-only ablations of the hybrid encoder. Experimental results demonstrate that TONet achieves an overall PCC of 0.67, a macro-AUROC of 0.82, and per-lead Bland-Altman biases below 0.003 mV in absolute terms on the physical voltage scale, outperforming the linear and mean-floor baselines by a large margin (Tables 1,2). To further verify that these gains are not specific to a single cohort, we also performed an independent in-distribution reconstruction evaluation on the Chapman-Shaoxing database (*n* = 43,559), where the identical TONet architecture, retrained from scratch, again exceeded the Kors-style baseline (PCC 0.612 vs. 0.498). These findings provide methodological support for further investigation of single-lead-to-multi-lead ECG reconstruction; the gap between the present in-distribution evaluation and real wearable deployment is discussed explicitly in the Limitations section.

**Table 1:** Per-lead bland-altman agreement and PCC on the physical voltage scale (mV).

Lead	Bias (mV) (95% CI)	LoA Lower (mV) (95% CI)	LoA Upper (mV) (95% CI)	Per-lead PCC (95% CI)
II	−0.0021 (−0.0029, −0.0014)	−0.2538 (−0.2578, −0.2498)	0.2495 (0.2455, 0.2534)	0.641 (0.630, 0.651)
III	−0.0018 (−0.0026, −0.0009)	−0.2578 (−0.2622, −0.2536)	0.2543 (0.2501, 0.2585)	0.640 (0.627, 0.651)
aVR	0.0024 (0.0020, 0.0029)	−0.1420 (−0.1460, −0.1392)	0.1468 (0.1438, 0.1511)	0.849 (0.842, 0.855)
aVL	0.0003 (−0.0001, 0.0007)	−0.1454 (−0.1483, −0.1425)	0.1460 (0.1431, 0.1491)	0.853 (0.847, 0.858)
aVF	−0.0014 (−0.0022, −0.0006)	−0.2491 (−0.2536, −0.2446)	0.2464 (0.2418, 0.2507)	0.512 (0.497, 0.525)
V1	−0.0022 (−0.0032, −0.0011)	−0.3309 (−0.3410, −0.3216)	0.3265 (0.3173, 0.3366)	0.693 (0.679, 0.707)
V2	−0.0014 (−0.0029, 0.0002)	−0.4969 (−0.5079, −0.4861)	0.4941 (0.4825, 0.5055)	0.691 (0.678, 0.703)
V3	−0.0018 (−0.0033, −0.0002)	−0.5071 (−0.5169, −0.4976)	0.5036 (0.4942, 0.5137)	0.646 (0.633, 0.659)
V4	−0.0005 (−0.0019, 0.0009)	−0.4479 (−0.4563, −0.4393)	0.4470 (0.4378, 0.4558)	0.655 (0.643, 0.667)
V5	0.0021 (0.0008, 0.0034)	−0.4039 (−0.4148, −0.3936)	0.4081 (0.3976, 0.4185)	0.659 (0.645, 0.673)
V6	0.0007 (−0.0007, 0.0021)	−0.4584 (−0.4980, −0.4197)	0.4599 (0.4195, 0.5017)	0.545 (0.512, 0.581)

**Table 2:** Ablation of architectural components and the multi-task objective (PTB-XL test fold).

Variant	CNN branch	Transformer branch	Classification head	Reconstruction head	Macro-AUROC (95% CI)	Overall PCC (95% CI)
Full TONet	✓	✓	✓	✓	0.821 (0.810, 0.831)	0.673 (0.648, 0.694)
Lead-I-only classifier	✓	✓	✓	✗	0.783 (0.771, 0.796)	—
CNN-only	✓	✗	✓	✓	0.747 (0.733, 0.761)	0.559 (0.531, 0.586)
Transformer-only	✗	✓	✓	✓	0.747 (0.734, 0.761)	0.624 (0.600, 0.647)
Reconstruction-only	✓	✓	✗	✓	—	0.663 (0.639, 0.685)

## 2 Materials and methods

### 2.1 Data source and preprocessing

This study utilized the PTB-XL database (12), currently one of the largest publicly available clinical electrocardiography datasets globally. The database contains 21,837 12-lead ECG recordings from 18,885 patients, with each recording lasting 10 s at a sampling frequency of 500 Hz. All data have been annotated by professional cardiologists according to SCP-ECG standards, covering a wide range of cardiac pathologies.

#### 2.1.1 Cohort splitting

To ensure objective evaluation and prevent data leakage, we strictly adhered to the dataset's recommended stratified splitting strategy. Specifically, folds 1–8 were allocated as the Training Set, fold 9 as the Validation Set, and fold 10 as the Test Set. This partition ensures that all records from the same patient appear in only one data subset. Regarding input feature construction, this study extracted only Lead I from the standard 12 leads as the model input as a controlled, in-distribution surrogate for single-lead acquisition. We emphasize that hospital-grade Lead I sampled at 500 Hz is not equivalent to true wearable Lead I, which is typically recorded at ≤250 Hz and contaminated by motion artefacts, electrode-placement variability, and different filtering pipelines; the implications of this distribution gap are addressed in the Limitations section. The complete 12-lead signals and their corresponding five major diagnostic classes (NORM, MI, STTC, CD, HYP) served as training targets.

#### 2.1.2 Data normalization

Given that different acquisition devices may cause signal baseline drift and amplitude discrepancies, we performed *Z*-Score normalization independently for each ECG recording. The calculation formula is as follows:

$$x^{\prime }=\frac{x-\mu }{\sigma +\epsilon }$$

where *x* is the original signal, μ and σ are the mean and standard deviation of the sample respectively, and ϵ is a small constant $\left(1\times 10^{-8}\right)$ to prevent division by zero. This preprocessing step eliminates the influence of amplitude scaling, allowing the model to focus more on learning waveform morphological features.

#### 2.1.3 Independent validation cohort (Chapman-Shaoxing)

To assess whether the proposed architecture generalises beyond a single clinical database, we additionally used the Chapman-Shaoxing 12-lead ECG database (33), which contains 45,152 records acquired from a separate population (Shaoxing People's Hospital, China) using different hardware. Signals are recorded at 500 Hz over 10 s, matching the temporal format of PTB-XL. We applied an automated quality-control pass (rejecting recordings with non-finite values, constant leads, or out-of-physiological-range amplitudes), retaining 43,559 recordings (96.5%). These were split 70/10/20 (*n*_train = 30,491; *n*_val = 4,356; *n*_test = 8,712) with a fixed random seed of 42 for reproducibility. Because Chapman is a rhythm-focused database whose SNOMED-CT taxonomy is not directly comparable to PTB-XL's SCP-ECG diagnostic super-classes, we restrict the Chapman evaluation to the reconstruction task—which is the core methodological contribution of TONet—and do not attempt cross-label harmonisation.

### 2.2 Model architecture

**Figure 1:**
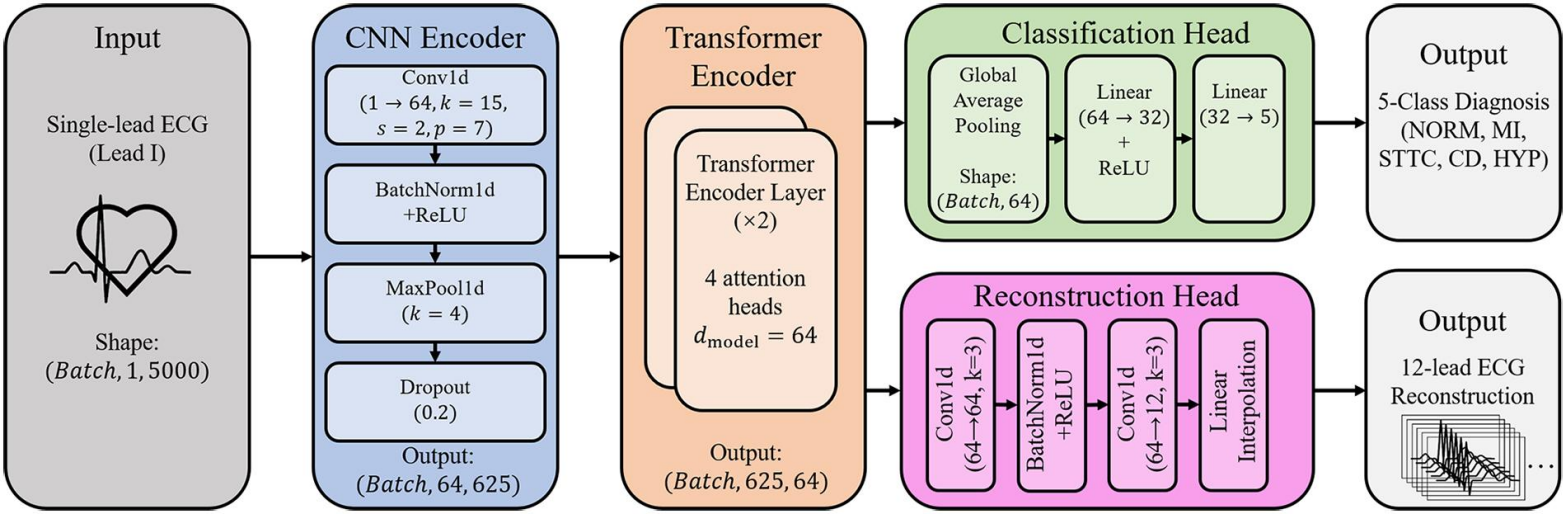
The overall architecture of the proposed TONet.

This study proposes a multi-task deep learning framework named TONet (Transformer-based Optimization Network). The architecture aims to balance local morphological feature extraction with global temporal dependency capture. Its core consists of three tightly coupled modules, as shown in Figure 1.

#### 2.2.1 Hybrid encoder

To address the limitations of traditional CNNs in modeling long sequences, we designed a serial “CNN-Transformer” encoding structure. First, data enters the CNN Feature Extraction Module, which contains a 1D convolutional layer with a large receptive field (Kernel Size = 15, Stride = 2), followed by batch normalization, ReLU activation, MaxPool (kernel = 4) and dropout (*p* = 0.2), designed to capture high-frequency morphological features such as rapid QRS complex changes and to downsample the temporal dimension from 5,000 to 625 tokens with an embedding dimension of d_model = 64. Subsequently, the feature map enters the Transformer Global Dependency Encoder. This layer contains two stacked Transformer Encoder Blocks utilizing a multi-head self-attention mechanism (Heads = 4, feedforward dimension = 256, pre-LayerNorm, GELU activation) to calculate correlations between any two token embeddings derived from the down-sampled signal (13). This mechanism enables the model to capture long-range dependencies spanning multiple cardiac cycles (such as the regularity of R-R intervals), thereby maintaining rhythmic consistency during reconstruction.

#### 2.2.2 Dual-task decoders

The model output consists of two parallel task branches:

##### 2.2.2.1 Reconstruction head

Utilizes two stacked 1-D convolutional layers (Conv1d, kernel = 3, padding = 1) with batch normalization and ReLU, followed by a linear-interpolation up-sampling step, to map the latent representation (Batch, 64, 625) back to the original resolution (Batch, 12, 5,000). We clarify here that the reconstruction head uses standard Conv1d layers with interpolation-based up-sampling, not learned transposed convolutions, which corrects an inconsistency between the figure and the text in the original manuscript.

##### 2.2.2.2 Classification head

Maps the Transformer output features, after Global Average Pooling, through a Multi-Layer Perceptron (MLP) to five diagnostic classes, outputting the predicted probability for each disease.

### 2.3 Loss function and training strategy

To preserve critical pathological features while ensuring waveform reconstruction accuracy, we designed a composite loss function $L_{\mathrm{total}}$:

$$L_{\mathrm{total}}=L_{\mathrm{cls}}+\lambda \cdot L_{\mathrm{recon}}$$

where $L_{\mathrm{cls}}$ employs Binary Cross-Entropy Loss to supervise the multi-label classification task, forcing the encoder to learn discriminative diagnostic features; $L_{\mathrm{recon}}$ employs Mean Squared Error (MSE) Loss to minimize the numerical difference between the reconstructed and ground-truth waveforms. Based on experimental validation, we set the weighting parameter λ to 2.0 to achieve the optimal balance between the two tasks.

### 2.4 Experimental setup and evaluation metrics

All models in this study were built using the PyTorch 2.4.1 framework and trained on a single NVIDIA GeForce RTX 4070 Laptop GPU (8 GB VRAM) under CUDA 12.6. Input recordings were processed without additional band-pass or notch filtering beyond the native PTB-XL filter chain; only per-recording *Z*-score normalization was applied (Section 2.1). We adopted the AdamW optimizer (14) (initial learning rate set to 1 × 10^−3^, weight decay set to 1 × 10^−4^) with default betas of $\;\beta_{1}=0.9$ and $\beta_{2}=0.999$ to improve convergence speed and prevent overfitting. The training process was set to up to 50 epochs with a batch size of 64, with early stopping triggered by a plateau in validation loss (patience = 5 epochs). To ensure full reproducibility, all experiments were initialized with a fixed random seed of 42. Within the Transformer module, no explicit sinusoidal or learned positional encoding was added, as the preceding CNN front-end inherently preserves and encodes relative local positional information. We utilized a constant learning rate schedule throughout training. Furthermore, no explicit class-reweighting strategy was applied to the binary cross-entropy loss, allowing the model to train on the natural prevalence of the PTB-XL dataset. Model performance was comprehensively evaluated using metrics across the following five dimensions:

1. **Signal Fidelity:** Uses the Pearson Correlation Coefficient (PCC) to measure the waveform similarity between reconstructed and real signals on a sample-by-sample basis.
2. **Clinical Agreement:** Uses Bland-Altman Analysis (15) to calculate the mean difference (Bias) and 95% Limits of Agreement (LoA) between real and reconstructed values to assess the reliability of amplitude reconstruction.
3. **Diagnostic Utility:** Calculates the Area Under the Receiver Operating Characteristic Curve (AUROC) to compare the performance differences in automated diagnosis when using real 12-lead vs. AI-reconstructed 12-lead ECGs.
4. **Ablation Components:** To isolate the contribution of each architectural element, we trained and evaluated four reduced variants on identical data splits: (i) a Lead-I-only classifier with the reconstruction head removed; (ii) a CNN-only variant with the Transformer encoder removed; (iii) a Transformer-only variant with the CNN front-end replaced by a stride-8 average-pool plus 1 × 1 projection; and (iv) a reconstruction-only variant with the classification head removed.
5. **Baseline Comparison:** We compared TONet against (i) a population-mean floor that predicts, at every time step, the per-lead mean waveform computed on the training set, and (ii) a Kors-style multivariate linear regression that maps the Lead I time series to each of the 11 missing leads on a point-wise basis.
6. **External In-Distribution Validation:** To verify that the reconstruction performance of TONet is not an artefact of training and testing on a single cohort, we additionally retrained the full TONet architecture from scratch on the Chapman-Shaoxing database using identical model hyper-parameters, identical optimiser, identical loss, and identical input/output format. For this evaluation only the reconstruction branch was trained (the classification branch was disabled), and all evaluation metrics (per-lead PCC, RMSE, Bias, 95% LoA) were computed using the same pipeline as for PTB-XL. No weights were transferred between datasets—this is a strict per-dataset in-distribution evaluation, not a cross-database generalisation test.
7. **Noise Robustness:** To probe the model's resilience to perturbations characteristic of wearable acquisition, we re-evaluated the trained TONet on test-set inputs corrupted with (a) additive Gaussian noise at signal-to-noise ratios of 10 dB, 5 dB and 0 dB, and (b) sinusoidal baseline wander at 0.5 Hz with amplitudes of 0.2, 0.5 and 1.0 (in normalized signal units). No retraining or test-time adaptation was performed.
8. **Statistical Inference:** To ensure the statistical rigor of our evaluation, standard error and uncertainty estimation were performed using non-parametric bootstrapping with 1,000 resamples to derive 95% confidence intervals (CIs) for all reported PCC and AUROC metrics. Furthermore, to rigorously demonstrate whether the proposed multi-task reconstruction-plus-classification pipeline significantly outperforms direct single-lead evaluation, a two-tailed DeLong test was executed across the multi-label evaluation output to compare the full TONet against the Lead-I-only baseline. A *p*-value of <0.05 was considered statistically significant.

## 3 Results

### 3.1 Signal reconstruction performance and model convergence

**Figure 2:**
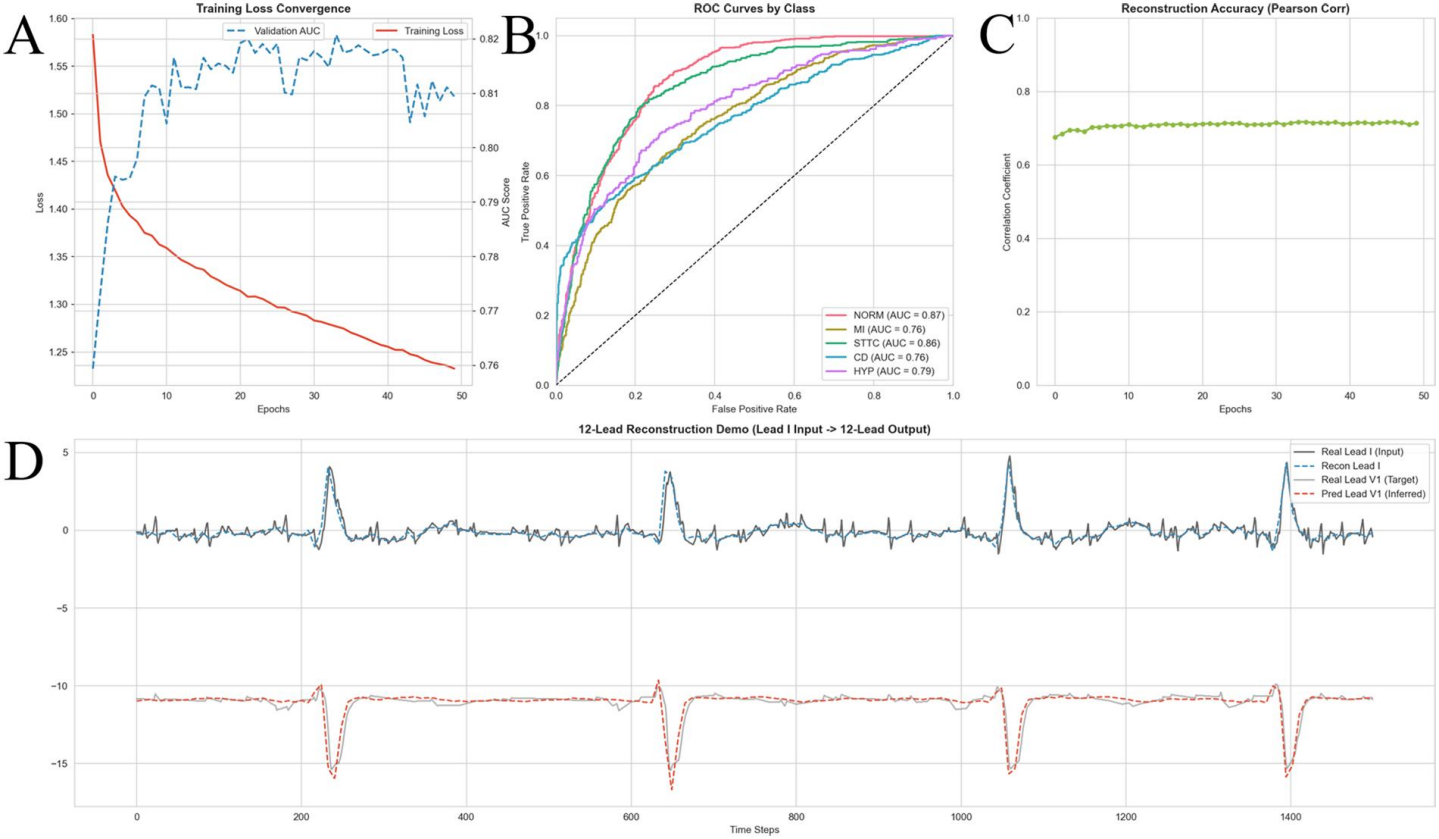
Model performance overview. **(A)** Training and validation loss convergence over 50 epochs. **(B)** Receiver Operating Characteristic (ROC) curves for the five diagnostic super-classes. The Area Under the Curve (AUC) indicates high diagnostic capability, particularly for Normal (0.87) and ST/T changes (0.86). **(C)** The progression of Reconstruction Accuracy (Pearson Correlation Coefficient) on the validation set, stabilizing around 0.673. **(D)** A snippet comparison of Lead I (input) and Lead V1 (inferred).

We evaluated the reconstruction performance of TONet on the independent PTB-XL test set (Fold 10). The loss convergence curve during training (Figure 2A) demonstrates that as the training epochs progressed, the Total Loss showed a steady downward trend. Moreover, the validation loss closely tracked the training loss decline without exhibiting significant overfitting, proving the model's robust generalization ability.

**Figure 3:**
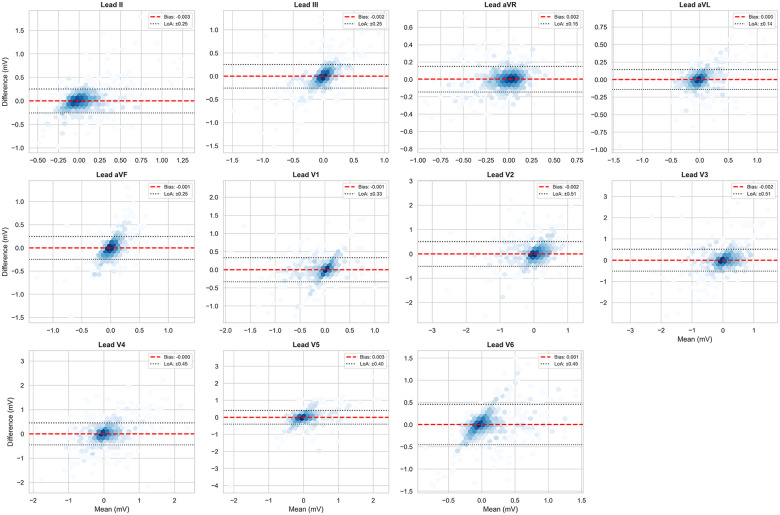
Per-lead bland–altman analysis of reconstructed 12-lead ECGs on the physical voltage scale.

Regarding signal fidelity, we utilized the Pearson Correlation Coefficient (PCC) as the core metric for measuring waveform morphological similarity. As shown in Figure 2C, the average PCC across all leads on the test set increased rapidly with training and eventually converged to an overall PCC of 0.673 on the held-out test set (Fold 10). This indicates that TONet can effectively leverage the temporal information from Lead I to infer the waveform trends of the other 11 leads; per-lead reconstruction quality is highest in the limb-derived leads (II, III, aVR, aVL, aVF) and lowest in the mid-precordial leads (V2–V3), as further detailed in the per-lead Bland–Altman analysis below (Figure 3 and Table 1).

### 3.2 Diagnostic efficacy assessment

Beyond waveform reconstruction, another core advantage of TONet lies in the diagnostic capability endowed by its multi-task learning architecture. We calculated the Area Under the Receiver Operating Characteristic Curve (AUC-ROC) for the model across five major diagnostic tasks; the results are presented in Figure 2B.

The model achieved a macro-averaged AUROC of 0.82 across the five super-classes, with the highest per-class score in the Normal (NORM) category, at AUC = 0.87, and in the clinically relevant ST-T change (STTC) category (typically associated with myocardial ischemia or electrolyte disturbances) at AUC = 0.86. This result is consistent with the interpretation that TONet does not merely “imitate” waveforms at the pixel level but also learns features with pathological relevance (such as subtle ST-segment deviations and T-wave inversions) through the classification branch of the multi-task objective. Even in the highly challenging Myocardial Infarction (MI) category, TONet achieved an AUC of 0.76 relying solely on single-lead input; however, as noted in the Limitations, this in-distribution AUROC value should not be interpreted as evidence of out-of-hospital screening performance.

### 3.3 Clinical agreement analysis

To further characterize the per-lead amplitude agreement of the reconstructed signals, we employed Bland-Altman agreement analysis. In contrast to the original submission, in which Bland-Altman statistics were inadvertently computed on *Z*-score-normalized signals, we now de-normalize each reconstruction back to the physical voltage scale (mV) using the per-recording mean and standard deviation, and report bias and 95% limits of agreement (LoA) separately for each of the 11 reconstructed leads.

The updated per-lead results are shown in Figure 3 and tabulated in Table 1. The bias is below 0.003 mV in absolute terms for every reconstructed lead, indicating no systematic over- or under-estimation. The 95% LoA are tightest in the augmented limb leads aVR and aVL (±0.14 mV), intermediate in the bipolar limb leads II, III and aVF (±0.25 mV), and widest in the precordial leads, where they range from ±0.33 mV at V1 to ±0.52 mV at V3.

These per-lead LoA are an order of magnitude smaller than the threshold underlying the Sokolow–Lyon criterion for left ventricular hypertrophy (*S*_V1_ + *R*_V5/V6_ ≥ 3.5 mV), and well below the Cornell voltage threshold (*R*_aVL_ + *S*_V3_ ≥ 2.8 mV in men, ≥2.0 mV in women) (16, 17). They are, however, on the same order as—or larger than—the 0.1 mV threshold used in the universal definition of ST-elevation myocardial infarction (STEMI). We therefore interpret these results as supporting the use of TONet-reconstructed signals for macroscopic voltage-based pattern recognition, but not for fine-grained STEMI discrimination; this point is discussed further in the Limitations section.

Bland–Altman agreement plots between ground-truth and TONet-reconstructed signals for each of the eleven reconstructed leads (II, III, aVR, aVL, aVF, V1–V6) on the held-out PTB-XL test fold (Fold 10, *n* = 2,158 recordings). For each lead, all sample points were first de-normalized from the *Z*-score domain back to the physical voltage scale (mV) using the per-recording mean and standard deviation, after which agreement statistics were computed on the resulting mV-scale signals. Each sub-panel plots the per-sample difference (true minus reconstructed amplitude, *y*-axis) against the per-sample mean of the two measurements (*x*-axis), both in mV. Density is encoded as a logarithmic hex-bin colormap (darker = higher local density), with 5,000 points sub-sampled per lead for clarity. The red dashed line marks the mean difference (Bias) and the two black dotted lines mark the upper and lower 95% Limits of Agreement (LoA = Bias ± 1.96 × SD); numerical values for each lead are inset in the upper-right corner of every panel and tabulated in Table 1. Across all eleven leads the bias remains within ±0.003 mV, indicating no systematic over- or under-estimation. The 95% LoA are narrowest in the augmented limb leads aVR and aVL (≈ ± 0.14 mV), intermediate in the bipolar limb leads II, III and aVF (≈ ± 0.25 mV), and widest in the mid-precordial leads V2 and V3 (up to ±0.52 mV), reflecting the larger spatial information gap between Lead I and the precordial plane. The bottom-right cell is intentionally left blank to preserve a 3 × 4 grid layout.

### 3.4 Ablation of architectural components and the multi-task objective

**Figure 4:**
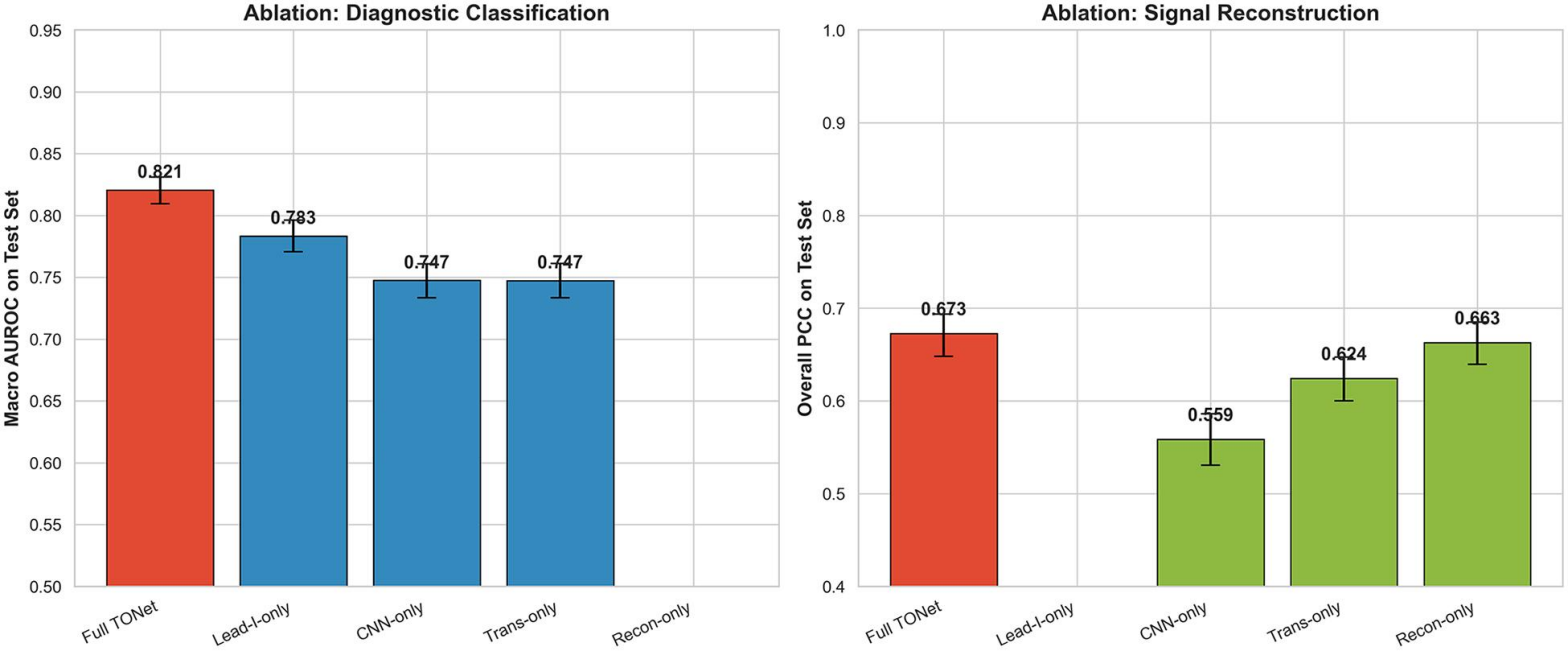
Ablation of architectural components and the multi-task objective on the PTB-XL test fold.

To isolate the contribution of each design choice, we evaluated four reduced variants on the same test fold as the full TONet (Figure 4, Table 2). Removing the reconstruction head and training the same encoder as a Lead-I-only classifier reduces macro-AUROC from 0.821 (full TONet) to 0.783, a 3.8-point drop, indicating that the dense waveform-reconstruction objective acts as a useful auxiliary supervision signal even for the classification task itself. Removing the Transformer encoder (CNN-only) reduces macro-AUROC to 0.747 and overall PCC from 0.673 to 0.559, while removing the CNN front-end (Transformer-only with a stride-8 average pool) yields a comparable 0.747 macro-AUROC and a PCC of 0.624. Removing the classification head (reconstruction-only) gives the highest pure-reconstruction PCC of 0.663 but, by construction, provides no diagnostic output. Taken together, these results indicate that the local-feature CNN front-end, the global-context Transformer, and the classification auxiliary loss each contribute non-trivially, and that none of the three can be removed without measurable performance loss.

### 3.5 Comparison with non-deep-learning baselines

To anchor the deep-learning results against an interpretable lower bound, we compared TONet with two non-deep-learning baselines on the same test fold (Table 3). A population-mean floor that simply outputs, at every time step, the per-lead mean waveform computed on the training set yields an overall PCC of −0.001, confirming that any model with substantially positive PCC is doing more than fitting the dataset mean. A Kors-style multivariate linear regression mapping Lead I to each of the 11 missing leads point-wise reaches an overall PCC of 0.492, capturing the dominant linear projection from frontal-plane to other-plane activity. The full TONet improves over linear regression by +0.18 absolute PCC (0.673 vs. 0.492). We interpret this margin as evidence that the value added by the deep network lies in modeling the non-linear, sequence-level dependencies that linear projection cannot represent, rather than in learning the dataset mean.

**Table 3:** Comparison with non-deep-learning reconstruction baselines (PTB-XL test fold).

Method	Type	Overall PCC
Population-mean floor	Constant per-lead training-set mean	−0.0007
Kors-style linear regression	Point-wise linear projection from Lead I	0.4921
TONet (this work)	Hybrid CNN–Transformer, multi-task	0.6733

### 3.6 Robustness to simulated wearable-style noise

Because TONet is trained on clinical-grade Lead I and not on true wearable recordings, we explicitly probed its degradation profile under two perturbations representative of consumer-device acquisition (Table 4). Under additive Gaussian noise at SNR = 10 dB the overall PCC is 0.686, slightly above the clean-signal value, with further degradation to 0.674 at SNR = 5 dB and 0.611 at the very aggressive SNR = 0 dB. Under sinusoidal baseline wander at 0.5 Hz, overall PCC is 0.705 at amplitude 0.2, 0.680 at amplitude 0.5, and 0.617 at amplitude 1.0 (in normalized signal units). Across both perturbations, performance degrades gracefully rather than catastrophically: even at noise levels well beyond typical clean-clinical conditions, the model retains a positive correlation that is comfortably above both the population-mean floor and the linear-regression baseline reported above. We stress that these are simulated perturbations and do not substitute for evaluation on real wearable recordings, which is discussed in the Limitations section.

**Table 4:** Robustness to simulated wearable-style perturbations (PTB-XL test fold).

Perturbation	Intensity	Overall PCC
None (clean reference)	—	0.6733
Additive Gaussian noise	SNR 10 dB	0.6864
Additive Gaussian noise	SNR 5 dB	0.6744
Additive Gaussian noise	SNR 0 dB	0.6109
Sinusoidal baseline wander (0.5 Hz)	Amp. 0.2	0.7053
Sinusoidal baseline wander (0.5 Hz)	Amp. 0.5	0.6804
Sinusoidal baseline wander (0.5 Hz)	Amp. 1.0	0.617

### 3.7 External in-distribution validation on an independent cohort

To assess whether the reconstruction performance reported above generalises beyond the single PTB-XL cohort, we conducted a strict per-dataset in-distribution evaluation on the Chapman-Shaoxing database (33) (Table 5). After quality control, 43,559 of 45,152 recordings (96.5%) were retained and split 70/10/20. The full TONet architecture was retrained from scratch using identical hyper-parameters; training converged smoothly over 25 epochs (validation MSE 0.525 → 0.479) without overfitting.

**Table 5:** Independent in-distribution validation on the chapman-shaoxing database (*n* = 8,712 test recordings).

Lead	PCC (95% CI)	RMSE (mV)	Bias (mV)	LoA Lower (mV)	LoA Upper (mV)
II	0.681 (0.642, 0.708)	0.128	0.002	−0.250	0.253
III	0.566 (0.528, 0.599)	0.124	0.001	−0.242	0.245
aVR	0.783 (0.702, 0.834)	0.087	−0.001	−0.172	0.17
aVL	0.777 (0.713, 0.813)	0.077	0.001	−0.150	0.151
aVF	0.556 (0.529, 0.582)	0.121	0.001	−0.236	0.238
V1	0.647 (0.617, 0.671)	0.168	0.001	−0.328	0.33
V2	0.661 (0.636, 0.687)	0.269	−0.004	−0.532	0.524
V3	0.635 (0.593, 0.665)	0.289	−0.003	−0.570	0.564
V4	0.639 (0.583, 0.679)	0.295	−0.000	−0.577	0.577
V5	0.652 (0.606, 0.691)	0.277	0.002	−0.540	0.544
V6	0.502 (0.462, 0.546)	0.322	0.002	−0.629	0.632
Overall (11 leads pooled)	0.612 (0.552, 0.666)	0.225	—	—	—
Linear-Regression baseline	0.498	—	—	—	—
Population-Mean Floor	0.01	—	—	—	—

On the held-out Chapman test fold (*n* = 8,712), TONet achieved an overall reconstruction PCC of 0.612 (95% CI: 0.552–0.666) and an overall RMSE of 0.225 mV, substantially outperforming both the Kors-style linear-regression baseline (PCC = 0.498) and the population-mean floor (PCC = 0.010). The relative ranking is preserved: in both datasets TONet exceeds the linear-regression baseline by more than 0.10 absolute PCC (PTB-XL: +0.181; Chapman: +0.114), while the two baselines themselves yield nearly identical values across the two datasets (mean-floor: −0.001 vs. +0.010; linear regression: 0.492 vs. 0.498), indicating that the comparison is fair and that the two cohorts present comparable intrinsic 1-to-12-lead reconstruction difficulty.

The per-lead Bland-Altman analysis on Chapman (Table 5) preserves the qualitative pattern observed on PTB-XL: agreement is strongest for the augmented limb leads (aVR PCC = 0.783; aVL PCC = 0.777), intermediate for the bipolar limb leads, and weakest for the inferior leads and V6 (PCC = 0.502), reflecting the inherent geometric difficulty of inferring leads that are nearly orthogonal to Lead I. Critically, the per-lead bias remains within ±0.005 mV for all eleven reconstructed leads, consistent with the absence of systematic voltage drift observed on PTB-XL. The 0.061 drop in overall PCC relative to PTB-XL (0.673 → 0.612) is reported transparently and is consistent with the larger rhythm-class spectrum of Chapman and somewhat higher signal-quality variability (1,593 recordings rejected by quality control, a higher rejection rate than for PTB-XL). Overall, the Chapman validation supports the conclusion that the architectural and training choices in TONet are not over-fitted to PTB-XL.

### 3.8 Visual case analysis

To visually illustrate the reconstruction effect on a representative case, we selected a patient (Patient ID: 1825) from the test set whose per-recording PCC was close to the upper tail of the test distribution for a visual comparison of the 12-lead reconstruction results; this sample achieved an overall correlation coefficient of 0.9108. This case is presented for qualitative illustration only and is not intended to be representative of average per-patient performance, which is more conservatively summarized by the test-set-wide PCC of 0.673 and the per-lead Bland-Altman analysis above.

**Figure 5:**
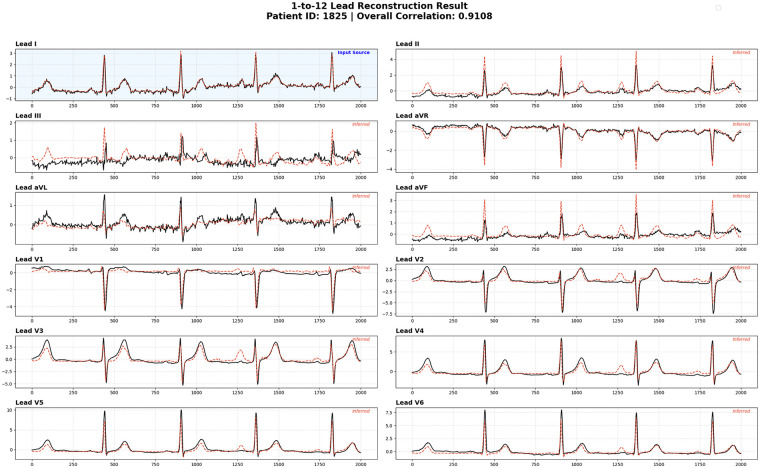
Representative 1-to-12 lead reconstruction result (patient ID: 1,825).

As shown in Figure 5, using Lead I as the sole input (blue shaded area), the other 11 leads generated by TONet (red dashed lines) coincide highly with the real recordings (black solid lines) in terms of overall rhythm and key waveform morphology. Notably:

1. **Recovery of R-wave Progression:** In precordial leads V1 to V6, the model accurately reproduced the physiological pattern of gradually increasing R-wave amplitude (R-wave progression).
2. **Preservation of Detailed Features:** In leads V2 and V3, the notching of the QRS complex and the morphology of the T-wave were well restored.

Although slight amplitude differences existed at the peak of the QRS wave in some leads (such as aVL), the gross morphological features of the reconstructed waveform (such as heart rate, rhythm, and frontal-plane axis estimates) remained broadly consistent with the reference recording. We refrain from claiming that this level of fidelity is sufficient for autonomous clinical interpretation, particularly for time-critical decisions such as STEMI triage.

## 4 Discussion

### 4.1 Key findings

This study provides a methodological evaluation of reconstructing a full 12-lead ECG from a single Lead I across two independent in-distribution clinical databases. Based on a hybrid CNN-Transformer architecture and a multi-task learning strategy, the TONet model achieved competitive in-distribution performance on the PTB-XL test fold. The average Pearson Correlation Coefficient across all leads reached 0.673, indicating that the reconstructed waveforms are broadly consistent with real recordings in terms of morphology. Per-lead Bland-Altman analysis on the physical voltage scale (Table 1) revealed a bias below 0.003 mV in every lead and 95% LoA between ±0.14 mV (aVL) and ±0.52 mV (V3). These LoA are well below the absolute voltage thresholds that define Sokolow–Lyon and Cornell criteria for left ventricular hypertrophy (≥2–3.5 mV) (16, 17), but on the same order as the 0.1 mV threshold for STEMI; we therefore interpret the amplitude-recovery results as supporting macroscopic voltage pattern analysis, not millimeter-level ischemia assessment.

Regarding diagnostic efficacy, TONet performed best in identifying Normal ECGs (AUC = 0.87) and detecting ST-T changes (AUC = 0.86), with a macro-averaged AUROC of 0.82 across the five super-classes. Given that ST-T changes are closely related to clinical conditions such as myocardial ischemia and electrolyte disturbances (18), these results indicate that the multi-task formulation preserves enough information in the latent representation to support the same coarse ECG super-class structure as the full 12-lead input. We deliberately refrain from extending this finding to out-of-hospital or pre-hospital cardiac event triage, since the present evaluation is entirely in-distribution and does not include any real-world prospective component.

Importantly, the independent in-distribution evaluation on the Chapman-Shaoxing database (Table 5) shows that these reconstruction-quality findings are not an artefact of training and testing on a single cohort. With no architectural change and no transferred weights, TONet achieved an overall PCC of 0.612 on Chapman vs. 0.498 for the linear-regression baseline—a margin closely comparable to the +0.181 PCC margin observed on PTB-XL. The qualitative per-lead pattern (strongest agreement for aVR/aVL, weakest for inferior leads and V6) is preserved across both cohorts, suggesting that the residual weak leads reflect the geometric information bottleneck of the single-lead input rather than dataset-specific artefacts.

### 4.2 Comparison with related work

Input Data Modality: From “Multi-Lead Assisted” to “True Single-Lead” Existing literature on “reduced-lead 12-lead ECG reconstruction” often overlooks a critical difference: the richness of the input data. A considerable amount of prior work, while claiming to “reduce leads,” utilizes inputs that still contain significant spatial vector information. For instance, the classic Kors regression method and its derivatives rely on Frank orthogonal vector leads (*X*, *Y*, *Z*), which already encompass the complete vector information of the heart's 3D electrical activity (19). Similarly, although the EASI lead system requires only five electrodes, its derived quasi-orthogonal leads also encode rich spatial localization information (20). The three-lead patch device used by Sohn et al. (7) also contains complementary information from different chest wall positions. From an information-theoretic perspective, the “reconstruction” in these methods is essentially closer to “coordinate transformation” or “spatial interpolation,” the difficulty of which is far lower than inferring all 12 leads from a single horizontal plane lead (Lead I)—the latter implies that the model must “guess” the electrical activity of V1–V6 based solely on temporal waveform features, with almost no spatial priors regarding the precordial leads.

One of the core contributions of this study is proving the possibility of high-quality reconstruction under this extreme constraint. We selected Lead I as the sole input, precisely simulating the standard ECG acquisition configuration of current consumer-grade smartwatches (e.g., Apple Watch, Samsung Galaxy Watch) (4). Despite the drastic reduction in available information, TONet achieved reconstruction effects comparable to or even better than multi-lead input methods through deep feature mining, fully demonstrating the effectiveness of the model architecture and the superiority of the algorithm.

#### 4.2.1 Model architecture evolution: transcending the limitations of traditional methods

From a methodological perspective, the TONet architecture represents an important paradigm shift in the field of ECG reconstruction, designed to directly address the core defects of prior methods.

##### 4.2.1.1 Contrast with pure convolutional neural networks

Early reconstruction models based on U-Net or ResNet (21, 22) excelled at extracting local waveform features but were constrained by the inherent limitations of convolutional operations—the receptive field size is determined by the kernel size, making it difficult to effectively capture long-range temporal dependencies spanning multiple cardiac cycles. This defect is particularly fatal in ECG signal processing: Heart Rate Variability (HRV), R-R interval regularity, and the temporal correlation between P-waves and T-waves all require the model to possess a “global view” for accurate modeling (23). In practice, pure CNN methods often lead to mismatches in the rhythm level of reconstructed waveforms, manifesting as poor temporal alignment of QRS complexes or phase shifts in T-waves.

##### 4.2.1.2 Contrast with generative adversarial networks

While GAN architectures (9) have achieved significant success in medical image synthesis, some researchers have attempted to apply them to ECG reconstruction tasks (8, 24). However, the “adversarial training” mechanism of GANs introduces a risk unacceptable in the medical field—Waveform Hallucination. To deceive the discriminator into generating “realistic-looking” waveforms, the generator may fabricate pathological features not present in the original signal (such as fake ST-segment elevation or abnormal Q-waves) or erase subtle abnormalities that actually exist. Such “creativity” might be a merit in artistic generation, but it is a catastrophic flaw in cardiovascular diagnosis where lives are at stake (25). Furthermore, the inherent instability of GAN training (mode collapse, vanishing gradients, etc.) further limits its reliability in clinical scenarios (26).

##### 4.2.1.3 TONet's solution

Addressing the aforementioned challenges, the TONet proposed in this study achieves a breakthrough through two key innovations. First, the introduction of the Transformer encoder fundamentally solves the “global view” problem—the self-attention mechanism enables the model to directly calculate the correlation between any two time points in the sequence, regardless of their distance (13). This characteristic allows TONet to simultaneously capture the instantaneous morphology of QRS complexes and rhythmic patterns across cardiac cycles, thereby maintaining temporal consistency during reconstruction. Second, the diagnostic classification task within the multi-task learning framework serves as an explicit pathological constraint, forcing the model to learn feature representations with clinical diagnostic significance rather than merely performing pixel-level fitting (11). This design ingeniously circumvents the “hallucination” risk of GANs: any fabricated waveform that does not conform to real pathological laws will be penalized in the classification task, thereby guiding the model to generate reconstruction results that are both morphologically realistic and pathologically reasonable.

### 4.3 Clinical implications

The present study should be read as a methodological step toward, rather than a clinical solution for, remote cardiac monitoring. Currently, the market penetration rate of global wearable ECG devices is growing rapidly (27), but the clinical value of these devices has long been constrained by the diagnostic limitations of single-lead ECGs—they can reliably detect arrhythmias and atrial fibrillation but cannot provide the spatial information required for myocardial ischemia localization (5, 6).

Within this framing, our results suggest two narrower potential use cases that warrant further, prospective investigation. First, the per-lead amplitude agreement reported in Table 1, with 95% LoA below 0.5 mV for the leads used in standard left-ventricular-hypertrophy voltage criteria, is consistent with—but does not by itself demonstrate—utility for macroscopic voltage screening at population scale, where the relevant decision thresholds are on the order of 2–3.5 mV (16, 17). Such use would still require explicit, prospective validation on cohorts with adjudicated LVH labels and on signals acquired from the intended wearable hardware. Second, the preservation of super-class diagnostic structure (macro-AUROC = 0.82) suggests that single-lead-derived 12-lead surrogates may have value as a low-cost pre-filter for downstream 12-lead workflows.

In contrast, we explicitly do not claim utility for STEMI triage, door-to-balloon time reduction (29), or infarct localization from single-lead input. STEMI diagnosis depends on millimeter-level ST-segment deviations of ≥0.1 mV (28), which is on the same order as—and at some leads smaller than—the per-lead LoA reported here. More fundamentally, regional pathology that is electrically silent on Lead I (for example, isolated posterior or right-ventricular infarction) cannot be recovered by any model that operates on Lead I alone; what such a model produces in the unseen leads is best understood as a population-prior imputation conditioned on Lead I, not as a patient-specific measurement (see Limitations).

### 4.4 Limitations and future directions

Despite the positive outcomes achieved in this study, several limitations need to be considered when interpreting the conclusions.

First—and most importantly—is the gap between simulated and real wearable acquisition. The training cohort of this study was a single hospital-grade database (PTB-XL (12)) acquired in a controlled environment at 500 Hz. This differs significantly from the usage scenarios of real wearable devices, which are typically recorded at ≤250 Hz and accompanied by noise sources such as motion artifacts, baseline drift, electrode-placement variability and poor electrode contact (30). We attempted to partially probe this gap by re-evaluating TONet under synthetic Gaussian noise and baseline wander (Table 4), and observed graceful degradation rather than collapse. In addition, the present revision includes an independent in-distribution evaluation on the Chapman-Shaoxing 12-lead database (33) (Table 5; *n* = 8,712 test recordings), where TONet, retrained from scratch using the identical protocol, achieved an overall reconstruction PCC of 0.612 and again outperformed the Kors-style linear-regression baseline by a margin closely comparable to that observed on PTB-XL (+0.114 vs. +0.181). This second-cohort in-distribution evaluation directly addresses the concern that the reported gains might be over-fitted to PTB-XL. Nevertheless, both databases consist of hospital-grade recordings; cross-database generalisation (training on one cohort and testing on another without retraining) and, more importantly, validation on cohorts recorded with actual smartwatch- or patch-based devices—for instance, the PhysioNet/CinC 2020 Challenge collection (34), including Ningbo, Georgia, CPSC2018 and CPSC-Extra—remain necessary next steps before any deployment claim can be sustained. We explicitly stress that synthetic noise and a second clinical database are not substitutes for real wearable signals: differences in front-end filtering pipelines, sampling rates, and electrode geometry introduce a distribution shift that no in-distribution evaluation can fully capture.

Second is the retrospective nature of the study design. This study employed retrospective cohort analysis and has not evaluated the impact of AI-reconstructed ECGs on actual clinical decision-making in prospective clinical trials, nor did any external clinician participate in the evaluation procedure of this work. Especially in any high-risk scenario, the clinical consequences of both false positives and false negatives cannot be ignored, necessitating strict diagnostic accuracy validation in real patient populations (31).

Third is the completeness of pathological coverage and the granularity of the label set. Although this study evaluated five major categories of ECG diagnoses (the PTB-XL “superclass” hierarchy), PTB-XL also provides finer-grained label sets (subclass, statement-level, rhythm and form) that we have not yet exploited; future revisions of this work will report performance under the more granular PTB-XL hierarchies. In addition, some rare but clinically important pathologies (such as channelopathies like Brugada syndrome and Long QT syndrome) were not fully validated due to sample size and class-imbalance limitations (32). The diagnosis of these diseases often relies on subtle waveform features in specific leads, and the reliability of TONet in such edge cases remains to be further studied. Because the PTB-XL super-class distribution is itself imbalanced, the macro-AUROC values reported here should be interpreted with appropriate caution and not as evidence of class-uniform diagnostic equivalence.

Fourth, a fundamental information-theoretic caveat applies to any single-lead-to-12-lead reconstruction approach, including TONet. Lead I records the projection of cardiac electrical activity onto a single frontal-plane axis. Pathology that is electrically silent or weakly represented on this axis—including, but not limited to, isolated right-ventricular infarction, isolated posterior infarction, and certain regional conduction disturbances—cannot be recovered from Lead I alone by any model. What a learned reconstructor produces in the unseen leads in such cases is best understood as a population-prior imputation conditioned on Lead I, i.e., the most likely waveform given the training-set distribution and the observed Lead I, rather than a patient-specific measurement. This implies an irreducible failure mode at the individual-patient level for rare, regionally localized pathology, regardless of the architecture or amount of training data, and it places a principled upper bound on the clinical claims that single-lead-to-12-lead reconstruction methods can support.

Fifth, we explicitly delineate the scope of clinical claims that the present results can support. The per-lead 95% LoA reported in Table 1 (±0.14 to ±0.52 mV) are well below the 2–3.5 mV thresholds used in standard LVH voltage criteria, but on the same order as—or in the case of V3 substantially larger than—the 0.1 mV threshold used in the universal definition of acute STEMI. We therefore explicitly do not claim utility of TONet-reconstructed signals for STEMI screening, ST-segment elevation quantification, or infarct localization. The appropriate near-term framing of this work is as a regularized methodological proof-of-concept for single-lead-to-multi-lead reconstruction, useful as a pre-screening or low-resolution voltage-pattern tool under explicit prospective validation, and not as a stand-alone diagnostic device.

Future research directions include: (1) conducting prospective external validation on cohorts collected by real wearable devices, since both PTB-XL and Chapman-Shaoxing remain hospital-grade databases; (2) designing prospective clinical trials to evaluate the actual utility of AI-reconstructed ECGs in emergency triage workflows; (3) exploring multi-modal fusion strategies by integrating common smartwatch sensor signals like Photoplethysmography (PPG) to further improve reconstruction accuracy; (4) developing lightweight model variants to achieve real-time On-Device inference, eliminating dependence on cloud computing; and (5) extending the present per-dataset in-distribution evaluation to a true cross-database generalisation protocol (e.g., train on PTB-XL, test on Chapman without retraining) to assess transferability without retraining.

## 5 Conclusion

This study proposes and validates TONet—a deep learning framework based on a hybrid CNN-Transformer architecture and a multi-task learning strategy—capable of high-fidelity reconstruction of full 12-lead ECGs from a single Lead I. Systematic evaluation on the PTB-XL dataset demonstrates that TONet exhibits encouraging performance across multiple dimensions, including signal fidelity, clinical agreement, and diagnostic efficacy. An independent in-distribution validation on the Chapman-Shaoxing database further confirms that the reconstruction benefit of the proposed architecture is preserved on a second, hardware- and population-distinct clinical cohort. Together, these results provide methodological support for the extended application of wearable devices in remote cardiac monitoring. Although there is still a considerable distance from laboratory validation to real clinical deployment, the findings of this study represent a key step towards the vision of “accessible hospital-grade ECG monitoring for everyone.”

## Data Availability

Publicly available datasets were analyzed in this study. This data can be found here: Repository: PhysioNetDataset: PTB-XL, a large publicly available electrocardiography dataset DOI: https://doi.org/10.13026/x4td-x982. Access Note: Due to patient privacy restrictions, the data are not fully open to the public. Access can be obtained by credentialed users who complete the required training (CITI Data or Specimens Only Research) and sign the Data Use Agreement on the PhysioNet platform. The Chapman-Shaoxing 12-lead ECG database is also publicly available on PhysioNet (https://doi.org/10.13026/wgex-er52).
